# Cyclin E overexpression as a biomarker for combination treatment strategies in inflammatory breast cancer

**DOI:** 10.18632/oncotarget.14689

**Published:** 2017-01-17

**Authors:** Angela Alexander, Cansu Karakas, Xian Chen, Jason P.W . Carey, Min Yi, Melissa Bondy, Patricia Thompson, Kwok Leung Cheung, Ian O. Ellis, Yun Gong, Savitri Krishnamurthy, Ricardo H. Alvarez, Naoto T. Ueno, Kelly K. Hunt, Khandan Keyomarsi

**Affiliations:** ^1^ Department of Experimental Radiation Oncology, The University of Texas MD Anderson Cancer Center, Houston, Texas, USA; ^2^ Department of Surgical Oncology, The University of Texas MD Anderson Cancer Center, Houston, Texas, USA; ^3^ Department of Pediatrics, Baylor College of Medicine, Houston, Texas, USA; ^4^ Department of Pathology, Stony Brook School of Medicine, Stony Brook, New York, USA; ^5^ University of Nottingham, School of Medicine, Nottingham, UK; ^6^ Department of Pathology, The University of Texas MD Anderson Cancer Center, Houston, Texas, USA; ^7^ Morgan Welch Inflammatory Breast Cancer Research Program and Clinic, Houston, Texas, USA; ^8^ Department of Breast Medical Oncology, The University of Texas MD Anderson Cancer Center, Houston, Texas, USA

**Keywords:** cell cycle, inflammatory breast cancer, CDK2, cyclin E, treatment

## Abstract

Inflammatory breast cancer (IBC) is a virulent form of breast cancer, and novel treatment strategies are urgently needed. Immunohistochemical analysis of tumors from women with a clinical diagnosis of IBC (*n* = 147) and those with non-IBC breast cancer (*n* = 2510) revealed that, whereas in non-IBC cases cytoplasmic cyclin E was highly correlated with poor prognosis (*P* < 0.001), in IBC cases both nuclear and cytoplasmic cyclin E were indicative of poor prognosis. These results underscored the utility of the cyclin E/CDK2 complex as a novel target for treatment. Because IBC cell lines were highly sensitive to the CDK2 inhibitors dinaciclib and meriolin 5, we developed a high-throughput survival assay (HTSA) to design novel sequential combination strategies based on the presence of cyclin E and CDK2. Using a 14-cell-line panel, we found that dinaciclib potentiated the activity of DNA-damaging chemotherapies treated in a sequence of dinaciclib followed by chemotherapy, whereas this was not true for paclitaxel. We also identified a signature of DNA repair–related genes that are downregulated by dinaciclib, suggesting that global DNA repair is inhibited and that prolonged DNA damage leads to apoptosis. Taken together, our findings argue that CDK2-targeted combinations may be viable strategies in IBC worthy of future clinical investigation.

## INTRODUCTION

Inflammatory breast cancer (IBC) is the most aggressive subtype of breast cancer, accounting for 2–5% of breast cancers in the United States but causing an estimated 10% of total breast cancer mortality. This mortality occurs despite the use of modern chemotherapy regimens given in the neoadjuvant setting including both anthracyclines and taxanes, followed by modified radical mastectomy and comprehensive post-mastectomy radiation [1–3]. This disproportional mortality and a low pathological complete response rate (of about 15% overall) indicate that the current treatment strategies for these patients are suboptimal, and more effective treatments are necessary [4]. One challenge in the current treatment of IBC is the lack of well-validated targets with available drugs, along with an incomplete understanding of the molecular basis of the relative chemoresistance that characterizes many IBC tumors. Gene expression and genomic characterization of breast cancer have revealed that a number of molecular subtypes exist that not only describe different underlying biology and therapeutic vulnerabilities but also result in disparate clinical outcomes [5]. For example, hormone receptor–positive cancers (which are mostly luminal A/B by PAM50 gene expression) are known to have more indolent biology than do triple-negative breast cancers (TNBCs), of which 80% are basal by PAM50 subtyping. Similar analyses of IBC samples by the World Consortium have confirmed the presence of all of these subtypes in IBC, albeit in a different distribution [6]. These results therefore have not uncovered a significant number of novel unique targets for evaluation in IBC.

One potential novel treatment strategy being investigated by several groups, including ours, is targeting CDK2. This follows logically from our discovery of tumor-specific isoforms of cyclin E that bind CDK2 more tightly and that promote its activity in a cell cycle–independent manner [7]. As a key regulator of the G1-S checkpoint, cyclin E acts as a potent oncogene driving aberrant proliferation. In addition to its role in regulating progression through the cell cycle, we have found additional oncogenic roles of cyclin E including centrosome reduplication, enhanced growth factor signaling, and increased cancer stem cell phenotypes [8–11].

Previously we have shown that overexpression of cyclin E in breast cancer patient samples is associated with poor prognosis [12]. In addition to overexpression of full-length cyclin E protein, tumors express low-molecular-weight isoforms, termed LMW-E, which are generated via amino terminus cleavage of full length cyclin E by the serine protease neutrophil elastase [7]. These LMW-E isoforms are sequestered in the cytoplasm where they regulate additional substrates not usually regulated by nuclear cyclin E/CDK2 complexes [13]. To understand the biological roles of LMW-E, we previously generated transgenic mouse models of mammary tumors driven by LMW-E in a setting of CDK2 proficiency or deficiency. CDK2 expression was necessary for mammary tumors driven by LMW-E, suggesting that cyclin E may serve as a biomarker for the use of CDK2 inhibitors [14]. In the mouse tumor model, roscovitine and meriolin 5 both significantly delayed the development of tumors. On the basis of these results, we hypothesized that breast cancer cells overexpressing cyclin E (including LMW-E) may be sensitive to CDK2 inhibitors either alone or in combination with other agents. We have recently reported that LMW-E is a poor prognostic biomarker in breast cancer, further providing rationale for investigation of this pathway; however, previous patient cohorts did not include patients with IBC [15, 16]. In this study, we sought to examine the relevance of the cyclin E-CDK2 pathway in human IBC samples and design targeted combination treatments targeting this pathway.

Currently two classes of selective CDK inhibitors are being developed clinically. One class includes specific CDK4/6 inhibitors (such as palbociclib, ribociclib, and abemaciclib), which are being used in cancers that retain Rb-pathway functionality and/or amplification of CDK4 or CDK6, such as ER-positive breast cancers, melanomas, and sarcomas [17, 18]. The other distinct class includes CDK inhibitors that are more selective for CDK1 and CDK2 as well as the transcriptional CDKs (CDK5, CDK7, and CDK9), and these drugs are being more widely studied in cancers including triple-negative breast cancer [19]. Dinaciclib (SCH727965) is a potent second-generation drug in this class that we have evaluated in both IBC and non-IBC breast cancer cell lines.

We found that IBC cell lines are sensitive to dinaciclib and to meriolin 5, another CDK2 inhibitor [20]. Because combination therapy is needed for aggressive cancers such as IBC, we next screened combinations of dinaciclib with other chemotherapy agents currently used clinically for the treatment of breast cancer, using a high-throughput cell survival assay we developed to examine pairs of drugs in both sequences to identify the most synergistic combinations to advance into preclinical and clinical trials. The expression of a panel of DNA repair genes following dinaciclib treatment in TNBC cell lines (IBC and non-IBC) significantly decreased. These results suggest that prolonged DNA damage as a consequence of decreased DNA repair may be the mechanism underlying the sequence-specific synergism with chemotherapies that cause DNA damage.

## RESULTS

### Cyclin E expression and localization in IBC tumors

**Figure 1:**
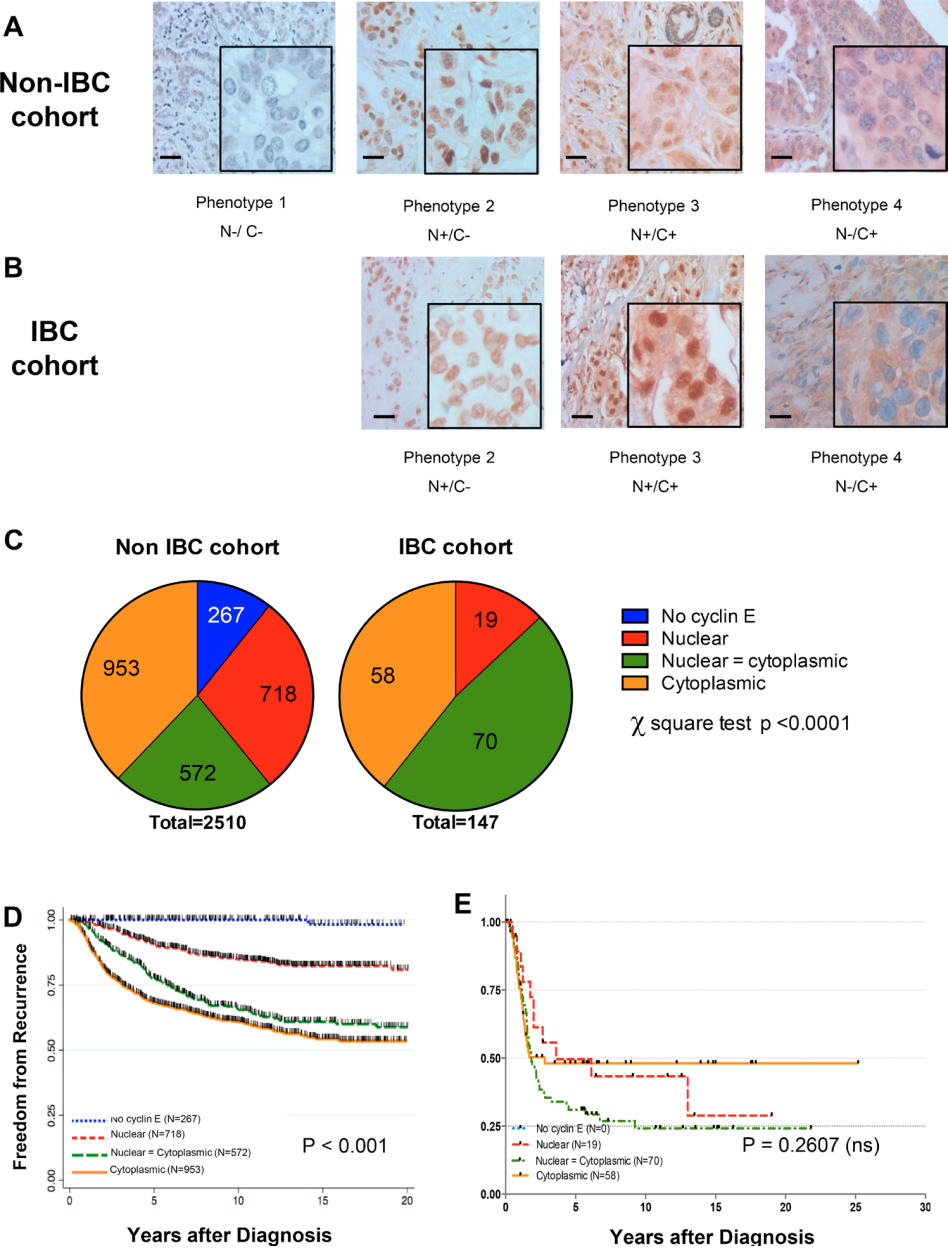
Cyclin E is overexpressed in IBC patient samples Representative immunohistochemical images showing cyclin E staining phenotypes in (**A**) non-IBC and (**B**) IBC tumors. N = nuclear staining C = cytoplasmic staining, either present (+) or absent (–) (**C**) Comparison of the distribution of staining phenotypes in non-IBC versus IBC cohorts. *P* < 0.0001, chi-squared test. (**D**, **E**) Kaplan-Meier survival plot showing the association between cyclin E staining (N and C and freedom-from-recurrence (FFR) for non-IBC cohort stratified by cyclin E phenotype regardless of hormone receptor/HER2 status. *P <* 0.0001. (E) Kaplan-Meier survival plot of non-IBC (D) and IBC (E) cohorts as a function of cyclin E phenotype.

To establish the clinical relevance of cyclin E as a therapeutic target in IBC, we examined the expression and localization of cyclin E in IBC samples from patients who received treatment or had consults at MD Anderson Cancer Center, using our previously established immunohistochemical method that incorporates both nuclear and cytoplasmic staining, and we compared these results to those for a large cohort of 2510 patients with non-inflammatory breast cancer [15, 16]. Clinical and treatment characteristics of all patient cohorts are shown in Table 1. Tumors were scored for cyclin E as one of four phenotypes, based on intensity and localization (Figure 1A): phenotype 1 tumors did not express significant cyclin E in the nucleus or cytoplasm, phenotype 2 tumors expressed nuclear cyclin E, phenotype 3 tumors expressed nuclear and cytoplasmic staining, and phenotype 4 tumors expressed cytoplasmic staining [16]. In our cohort of 2510 non-IBC patients, 11% of the samples had no cyclin E staining, 28% had nuclear positivity, 23% had nuclear and cytoplasmic positivity, and 38% had cytoplasmic staining (Figure 1A, 1C). On the other hand, the distribution of nuclear and cytoplasmic cyclin E staining was very different in the IBC cohort, where we observed 100% positivity for cyclin E in all the samples examined. Only 13% of the IBC samples had only nuclear staining, whereas the remaining 87% were strongly positive for cytoplasmic cyclin E (48% had nuclear and cytoplasmic staining and 39% had cytoplasmic staining) (Figure 1B, 1C).

**Table 1:** Clinical variables for patient samples

	Non-IBC (*N* = 2510)	IBC (*N* = 147)	*P* value
Age at diagnosis (year)			< 0.0001*
Mean	61.1	49.8	
Median (Range)	62 (25–96)	49 (23–76)	
< 50, no. (%)	629 (25.1)	76 (51.7)	< 0.0001
> = 50, no (%)	1881 (74.9)	71 (48.3)	
Tumor category, no (%)			< 0.0001^
T1	1282 (51.2)	0	
T2	1182 (47.2)	0	
T3	23 (0.9)	0	
T4	15 (0.6)	147 (100)	
Unknown	8		
Nodal status, no. (%)			< 0.0001
Negative	1533 (61.4)	0	
Positive	962 (38.6)	147 (100)	
Chemotherapy, no (%)			< 0.0001
No	1672 (66.6)	0	
Yes	838 (33.4)	147 (100)	
Radiation therapy, no (%)			< 0.0001
No	1616 (64.4)	32 (21.8)	
Yes	894 (35.6)	115 (78.2)	
Adjuvant Endocrine therapy, no (%)			0.02
No	1363 (54.3)	95 (64.6)	
Yes	1147 (45.7)	52 (35.4)	
Stage, no. (%)			< 0.0001^
I	1138 (45.3)	0	
II	1176 (46.8)	0	
III	190 (7.6)	131 (89.1)	
IV	0	16 (11.9)	
Unknown	6		
Tumor grade			< 0.0001^
I	324 (16.4)	0	
II	990 (49.9)	32 (22.2)	
III	668 (33.7)	112 (77.8)	
Unknown	528	3	
Estrogen receptor status			< 0.0001^
Negative	693 (28.1)	60 (45.1)	
Positive (> 1% cutoff)	1772 (71.9)	73 (57.9)	
Unknown	45	14	
Progesterone receptor status			< 0.0001^
Negative	949 (38.5)	78 (58.6)	
Positive (> 1% cutoff)	1514 (61.5)	55 (41.4)	
Unknown	47	14	
HER2 status			< 0.0001^
Negative	2113 (85.3)	63 (61.2)	
Positive	365 (14.7)	40 (38.8)	
Unknown/not tested	32	44	
Cyclin E phenotypes			< 0.0001
1	267 (10.6)	0	
2	718 (28.6)	19 (12.9)	
3	572 (22.8)	70 (47.6)	
4	953 (38)	58 (39.5)	

*Wilcoxon rank-sum (Mann-Whitney) test; ^*p* value calculated after excluded unknown category.

Cytoplasmic staining of cyclin E in the non-IBC cases was significantly correlated with poor prognosis (*P* < 0.001, Figure 1D), whereas all patients in the IBC cohort had a poor outcome (Figure 1E), regardless of nuclear or cytoplasmic expression of cyclin E. These results suggest that expression of any cyclin E is likely to be an essential oncogenic driver for IBC pathogenesis, and we reason that the high frequency of overexpression makes this pathway an ideal target for therapy.

### Targeting cyclin E in IBC and non-IBC cell lines

**Figure 2:**
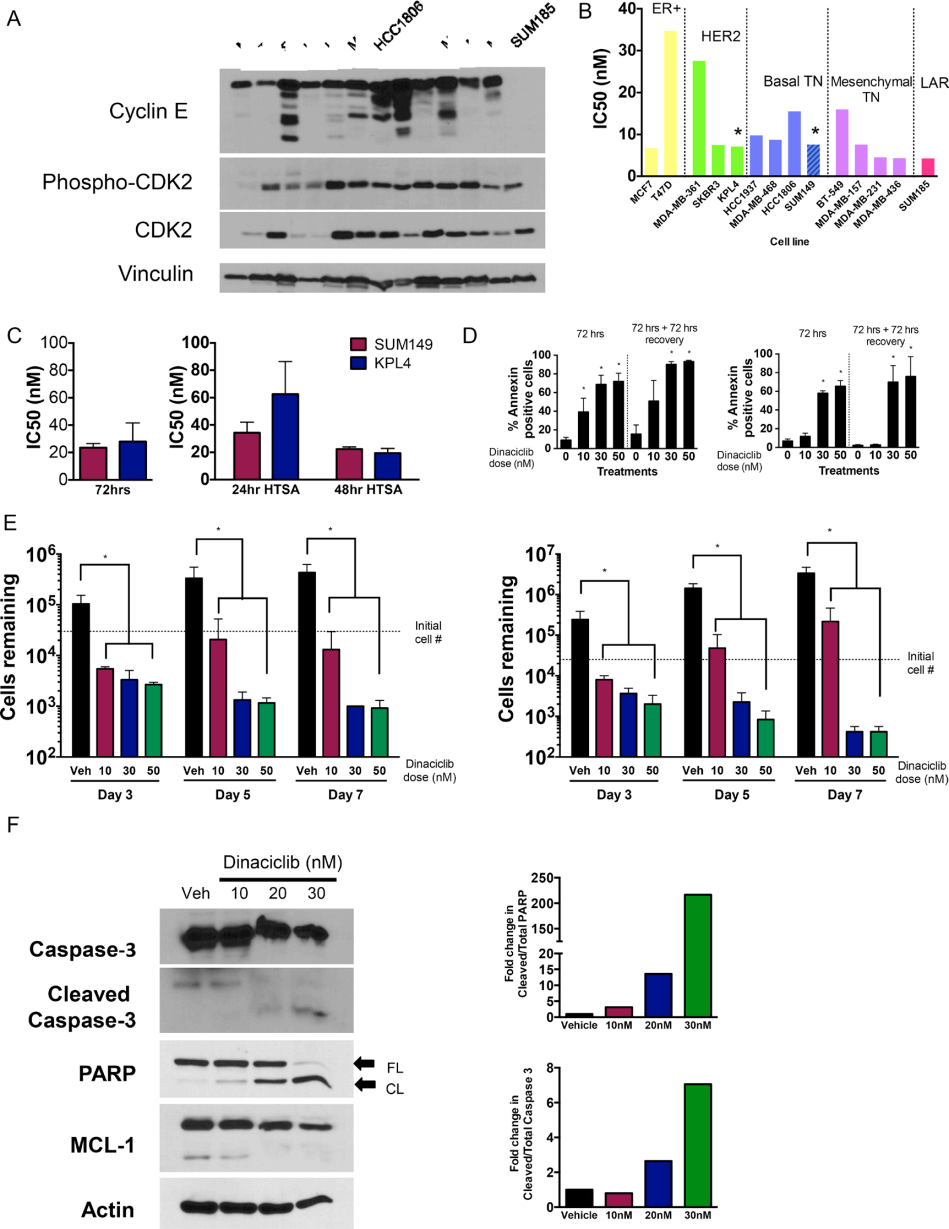
CDK2 is a target in breast cancers including IBC (**A**) Western blot of 13 breast cancer cell lines including IBC cells (SUM149 and KPL4) showing activation of cyclin E/CDK2 pathway particularly among TNBC lines. (**B**) IC_50_ values of dinaciclib (12 day assay) in 13 breast cancer cell lines. Bars are color-coded by molecular subtype, with both basal-like subtypes combined and both mesenchymal subtypes combined. Asterisks refer to the two IBC cell lines. (**C**) IC_50_ values for IBC cell lines treated with meriolin 5 for either 72 hours (left graph) or 24/48 hours and allowed to recover for 12 days prior to the MTT assay (right graph). (**D**) IBC cells (left panels, SUM149 cells; right panels KPL4 cells) were treated with indicated concentration of dinaciclib for 72 hours and subjected to Annexin V staining at the end of treatment or 72 hours post treatment. *P < 0.05 compared to 0 control. (**E**) IBC cells (left panels, SUM149 cells; right panels KPL4 cells) were treated with indicated concentration of dinaciclib for 3, 5 and 7 days and subjected to cell proliferation assays. *P < 0.05 compared to DMSO control. Error bars: standard deviation. (**F**) Western blot showing increase in apoptosis markers (cleaved caspase 3 and cleaved PARP), and downregulation of Mcl1. Densitometry analysis of cleaved PARP and caspase 3 are depicted in the graphs on the right. β-actin serves as loading control for gels.

We next investigated whether treatment of IBC cell lines (SUM149 and KPL4) with CDK inhibitors is a viable therapeutic option. SUM149 is a BRCA1-deficient triple-negative IBC cell line, and KPL4 is a HER2-overexpressing (but trastuzumab-resistant) cell line. These models were chosen as established models that grow well in 2-dimensional culture and at sufficiently low density for our long-term assays. Both established lines had high levels of full-length cyclin E, and SUM149 also expressed LMW-E isoforms and higher phospho-CDK2 (Thr160) expression compared with KPL4 (Figure 2A).

Dinaciclib, a potent CDK2 inhibitor (as well as CDK1, CDK5, and CDK9 inhibitor) that is currently in clinical trials for several cancers, was used to target the cyclin E/CDK2 pathway. We compared the IBC cell line sensitivity to dinaciclib to that of a panel of 12 other breast cancer cell lines from all molecular subtypes including the Lehmann TNBC subtypes except for immunomodulatory (Supplementary Table 1 and 2) [21]. Dose-response analysis of dinaciclib indicated that all IBC and non-IBC breast cancer cell lines (with the exception of T47D) were highly sensitive to dinaciclib, with half maximal inhibitory concentration (IC_50_) values ranging from 4.24 nM to 18 nM following 24-hour treatment (Figure 2B, Supplementary Table 3). We also examined meriolin 5, a structurally distinct CDK2 inhibitor [20], and found that the IC_50_ values of meriolin 5 in both IBC cell lines were in the low nanomolar range (between 20 and 80 nM) depending on treatment time and assay length; however, 72 hours of exposure was enough to cause maximal cytotoxicity (Figure 2C). Taken together, these results suggest that CDK2 is a valid target in IBC worth further consideration.

To determine whether dinaciclib induced apoptosis, we performed annexin V/PI staining and also counted the cell numbers as a function of days after drug treatment (day 3) and at 2 and 4 days after drug removal. We observed a dose-dependent increase in annexin V positive cells at both time intervals in both IBC cell lines, and cell death continued even after drug removal (Figure 2D). These results corresponded with the significantly decreased cell numbers at all concentrations of dinaciclib, supporting the conclusion that dinaciclib has direct cytotoxic activity in IBC cells (Figure 2E). Dinaciclib treatment also increased cleaved PARP and cleaved caspase 3, additional indicators of apoptosis. We also confirmed that Mcl1 is downregulated in response to dinaciclib treatment, consistent with previous findings in other systems (Figure 2F).

### Combination treatment with CDK2 inhibitors in IBC

**Figure 3:**
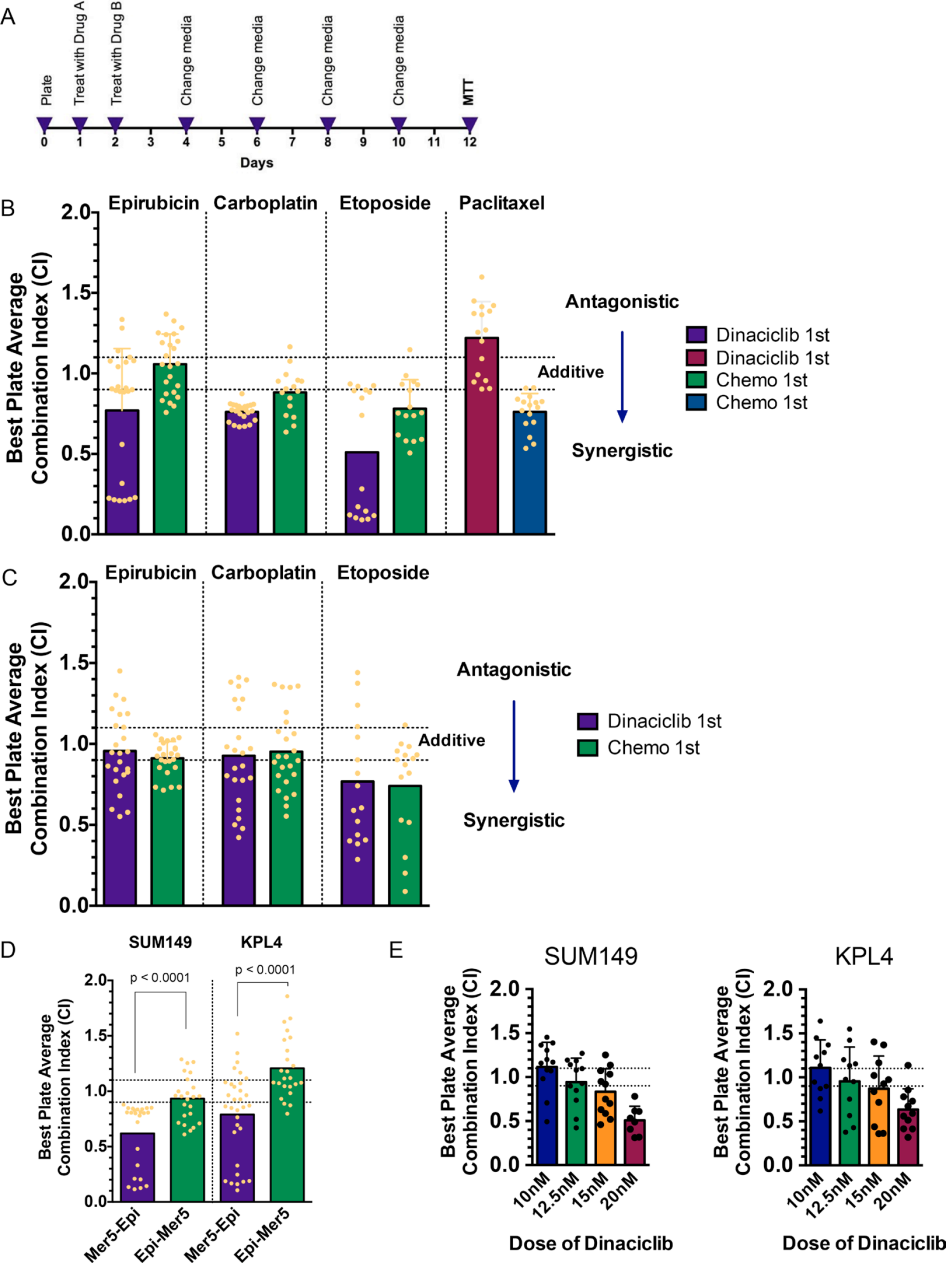
Novel combination strategies involving CDK2 inhibitors with chemotherapy/radiation (**A**) Schematic for high-throughput survival assay (HTSA). Cells are plated at pre-defined densities in 96-well plates 24 hours prior to beginning drug treatments. The two drugs (arbitrarily called drug A and drug B) are added 24 hours apart, with the medium changed on day 2 and every 48 hours there after. Drug A concentrations are IC_25_, IC_50_, and IC_75_, and drug B concentrations range from IC_10_ through IC_60_, of which there are eight combinations of doses per plate. The MTT assay is performed on day 12 as a readout of cell survival fraction. Survival fraction data are input into CalcuSyn software to generate combination indexes (CIs). (**B**, **C**) CI of sequential treatment of SUM149 cells (B) and KPL4 (C) with dinaciclib followed by the indicated chemotherapies (purple and maroon bars) or the indicated chemotherapies followed by dinaciclib (green and blue bars). Each dot represents one of the pairs of drug concentrations. Dotted lines represent the CI range of additivity (0.9–1.1), antagonism (> 1.1) or synergism (< 0.9). (**D**) CI of sequential combination treatment of meriolin 5 followed by epirubicin (purple bars) or epirubicin followed by meriolin 5 (green bars) in SUM149 and KPL4 cells. (**E**) CI of sequential combination treatment of dinaciclib followed by radiation (as “drug B”). SUM149 cells received 1, 2, 4, or 6 Gy of radiation, and KPL4 cells received 3, 6, 9, or 12 Gy of radiation. The dotted lines represent the CI range of additivity as described in panels B and C.

We next set out to identify treatment strategies to improve upon the activity of dinaciclib. To this end, we evaluated whether sequential combinations of dinaciclib and different classes of chemotherapy that are being used or have been used in breast cancer act in a synergistic manner. To do this in a high-throughput comprehensive way, we designed a 96-well plate format high-throughput survival assay (HTSA) [22]. This assay, which can be used for any adherent cell line, allows us to analyze the combination of two drugs added sequentially or concomitantly and to examine the survival fraction after an extended period of time in culture (12 days), similar to how a clonogenic assay is performed (Figure 3A). The drug concentrations used for these experiments are shown in Supplementary Tables 3, 4, 5, 6, 7, 8. The survival fractions for each of the 24 pairs of drug A-B concentrations were calculated, and CalcuSyn software was used to derive combination indexes (CIs) for each using the Median Effect models described by Chou and Talalay [23]. The absolute value of the CIs determines the degree of synergy or antagonism of drug combinations, and we considered CIs below 0.9 to be synergistic and above 1.1 to be antagonistic.

We focused on using both IBC cell lines to comprehensively investigate a combination of dinaciclib with chemotherapy drugs (epirubicin, carboplatin, etoposide, and paclitaxel) in both sequences. In SUM149 cells, we found that dinaciclib followed by epirubicin, carboplatin, or etoposide demonstrated moderate to strong synergy, whereas the reverse sequence was either additive or less synergistic (Figure 3B and Supplementary Table 9). However, dinaciclib followed by paclitaxel was antagonistic in this cell line, while the reverse sequence was slightly synergistic. Paclitaxel acts as an anti-mitotic chemotherapy via tubulin polymerization and blocking depolymerization of microtubules, whereas the other three chemotherapy drugs tested all have DNA damage as a key mechanism of action (whether via direct DNA adducts or via inhibition of topoisomerase activity). We confirmed the result that dinaciclib followed by DNA-damaging chemotherapy is effective in KPL4 cells; unlike in SUM149 cells, however, in KPL4 cells the sequence did not matter as much to the synergism observed (Figure 3C). To confirm these results with a different CDK2 inhibitor, we used meriolin 5 in place of dinaciclib and determined the combination synergism using the same HTSA method. Consistent with the previous result, meriolin 5 induced moderately strong synergy (CI = 0.62 for SUM149 and CI = 0.79 for KPL4), whereas the reverse sequence was additive or antagonistic (CI = 0.93 for SUM149 and CI = 1.21 for KPL4) (Figure 3D).

Given that we observed that CDK inhibition potentiates DNA-damaging agents in IBC, we reasoned that dinaciclib may also act as a radiosensitizer if given prior to radiation. To investigate this, we adapted the HTSA to allow for radiation to be “drug B” and once again tested this combination in both IBC cell lines. SUM149 cells received 1–6 Gy and KPL4 received 3–12 Gy of radiation after treatment with 10–20 nM dinaciclib. These results demonstrated that, as the dose of dinaciclib increased, so did the magnitude of synergy, starting from additivity at a low dose (10 nM) and showing strong synergy at 20 nM, the highest dose tested (Figure 3E).

### Predictors of synergism in IBC and non-IBC models

**Figure 4:**
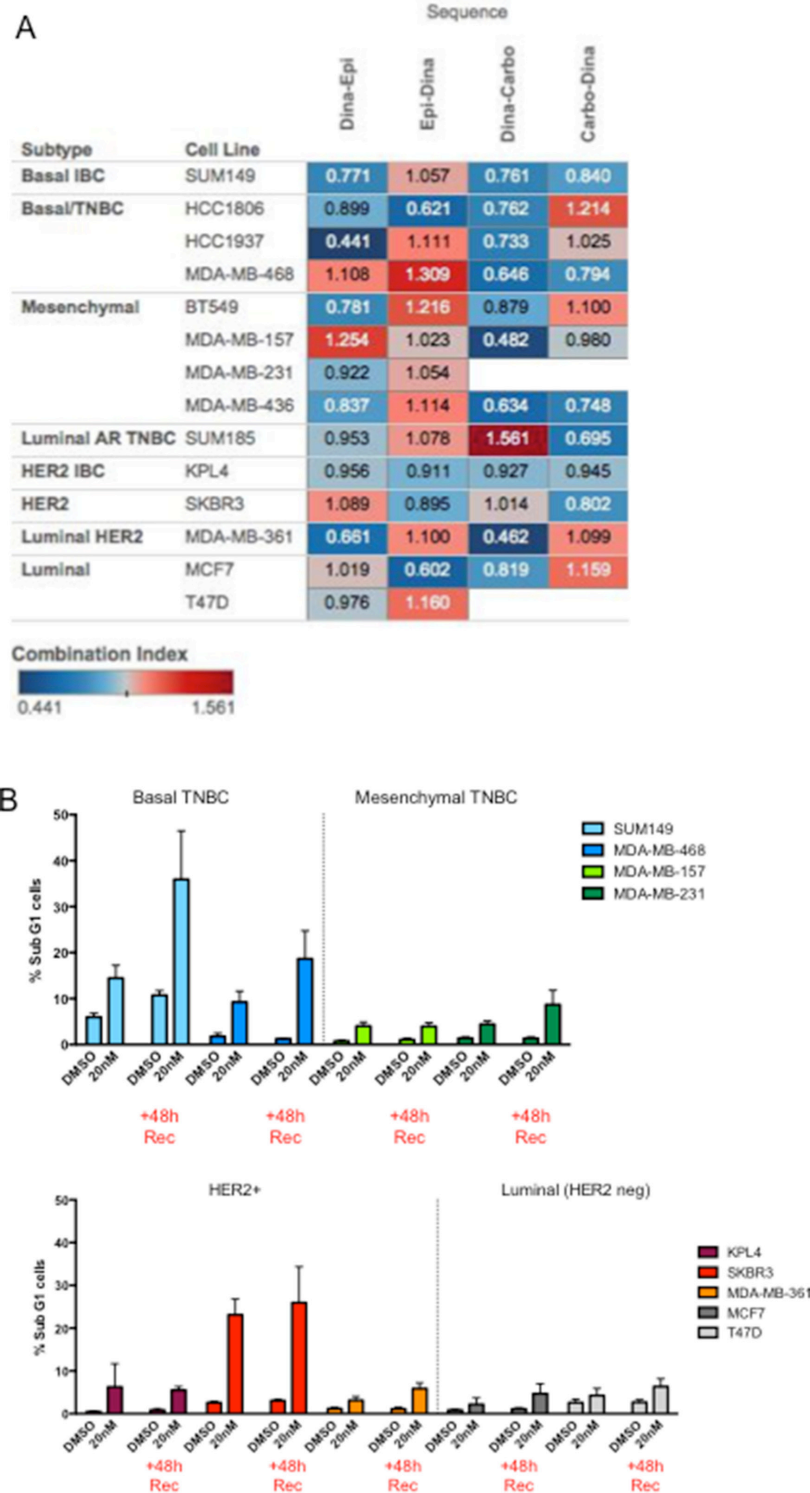
Predictors of response to dinaciclib/combination treatment (**A**) Heatmap of CIs representing a comprehensive 14–cell line panel screen, using 4 different sequential drug combinations: dinaciclib (Dina) with either epirubicin (Epi) or carboplatin (Carbo) in both sequences. Blue indicates synergism and red indicates antagonism. (**B**) The indicated cells (color coded for each subtype) were treated with DMSO or 20 nM dinaciclib for 48 hours. Cells were subjected to flow cytometry at the end of the treatment period of 48 hours post treatment (+48 h Rec) to measure sub G1 population.

We next examined the generality of the sequence-specific synergism of dinaciclib followed by different classes of chemotherapeutic agents in non-IBC cell lines. Molecularly, IBC cells are similar to subtype-matched non-IBC cells, and even within the TNBC subtypes, the distribution of subtypes among TNBC and TN-IBC is similar when applying the Lehmann classification [21]. We used a total of 14 cell lines from different subtypes for this analysis (Figure 4A). Using a cut-off of ≤ 0.9 to define synergy, we found five of the 14 cell lines (SUM149, HCC1937, BT549, MDA-MB-436, and MDA-MB-361) were synergistic when dinaciclib was “drug A” and epirubicin was “drug B,” and in all five lines the average CI was higher for the reverse sequence. Similarly for carboplatin as “drug B,” we found that eight of the 14 cell lines (SUM149, HCC1806, HCC1937, MDA-MB-468, BT549, MDA-MB-157, MDA-MB-436, and MCF7) showed even more synergy in general compared with epirubicin, and once again in all of these lines the average CI was higher for the reverse sequence (Supplementary Tables 9 and 10).

Since we examined the synergistic response of four different combination strategies in 14 different cell lines, we next set out to identify predictors of response. Specifically, we asked whether molecular subtype, p53 status, or the ability of cells to arrest in G1 or G2/M could predict response to the combination of dinaciclib and chemotherapy. However, none of these factors provided any predictive value to the synergistic response (Supplementary Figure 2A–2C). For example, the majority of TNBC tumors (85%) and all TNBC cell lines examined in this study harbor a mutation in the TP53 gene. It is plausible that loss of the G1 checkpoint as a result of p53 loss of function would poise tumor cells for subsequent G2 checkpoint-targeting agents. Given that our data shows that the mechanism of dinaciclib-induced synergy involves a widespread downregulation of DNA repair genes and master transcriptional regulators, it is unlikely for p53 status to be critical to synergy. However, when we analyzed the proportion of sub-G1 cells both after 48 hours of treatment and after 48 hours of recovery in fresh medium and stratified the cells by subtype, we found that both basal-like cell lines (SUM149 and MDA-MB-468) and one HER2+ cell line (SKBR3) had significant increases in sub-G1 following treatment, which increased in the 48-hour recovery set, suggesting that cell death continued to be induced even after drug removal (Figure 4B).

### Dinaciclib-induced DNA damage in the synergistic cell lines

**Figure 5:**
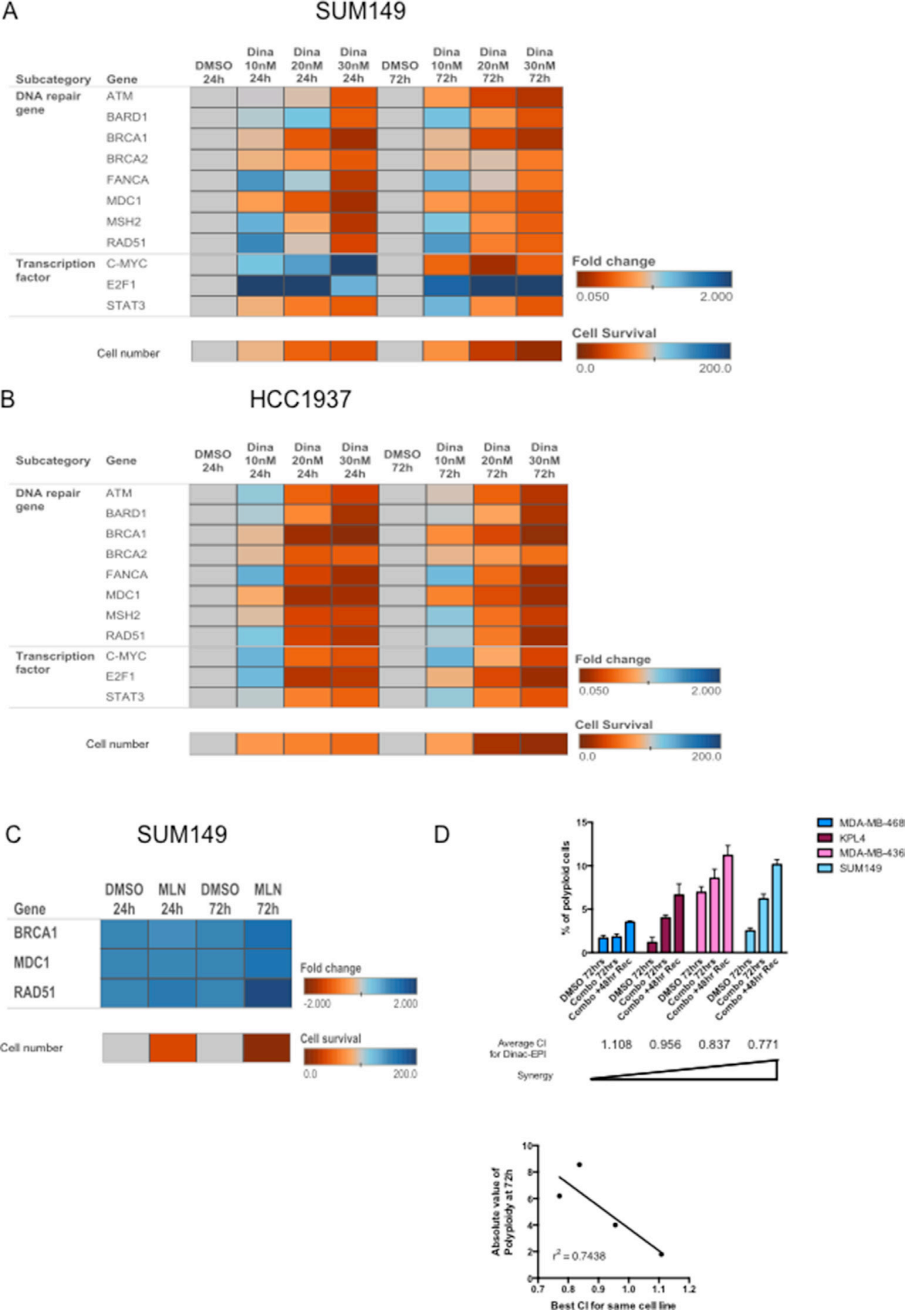
Dinaciclib inhibits multiple DNA repair pathways to induce synergism with DNA-damaging chemotherapy and induce polyploidy (**A**, **B**) Heatmap depicting fold changes for expression of the indicated genes (as measured by qRT-PCR) in SUM149 (A) or HCC1937 (B) cells treated with 10–30 nM dinaciclib for 24 or 72 hours. Fold changes were compared within each time point, compared with DMSO control. The bottom row indicates cell number (as a percentage of control) for each condition, to correlate the magnitude of change in gene expression with cytotoxicity of the drug. (**C**) Heatmap depicting fold changes for expression of the indicated genes (as measured by qRT-PCR) in SUM149 cells treated with 1 μM MLN8237 for 24 or 72 hours. The cell number row was calculated similarly to A. (**D**) Cells were treated with sequentially with dinaciclib followed by epirubicin with or without a 48 hour recovery period and subjected to polyploid DNA content by FACS analysis. SUM149 (8 nM dinaciclib and 20 nM epirubicin), KPL4 (10 nM dinaciclib and 15 nM epirubicin), MDA-MB-468 (18 nM dinaciclib and 5 nM epirubicin), and MDA-MB-436 (12.52 nM dinaciclib and 4 nM epirubicin). The average CI values for the dinaciclib-epirubicin combination are listed under each cell line bars. Bottom panel: Concordance of CI and polyploidy as depicted in the logistic regression analysis graph. Error bars represent standard deviation based on two or three independent experiments.

Because both radiation and chemotherapies such as epirubicin and carboplatin induce DNA damage, and DNA repair induction is necessary for cell survival [24–32], we next investigated whether dinaciclib treatment of cells (as a single agent) can also modulate DNA damage checkpoints to inhibit repair of chemotherapy-induced DNA damage. To this end, IBC and non-IBC cells were treated with 10–30 nM dinaciclib and subjected to qRT-PCR with a panel of genes involved in DNA damage checkpoints (ATM, BARD1, BRCA1, BRCA2, FANCA, MDC1, MSH2, and RAD51) [24–32]. These genes are transcriptionally regulated by several transcription factors including c-Myc, E2F1, NF-κB, and STAT3, which we also examined. In both SUM149 and HCC1937 cells, we observed two patterns of gene expression: a subset was downregulated at all doses (ATM, BRCA1, BRCA2, MDC1), whereas the other genes (BARD1, FANCA, MSH2, RAD51) showed a biphasic response in that at low doses they were increased but at higher doses (sufficient for pronounced cell growth inhibition) the genes were downregulated (Figure 5A and Supplementary Table 11). These gene expression results held true for both 24 and 72 hours of dinaciclib treatment (Figure 5B and Supplementary Table 12).

Examination of the transcriptional factors known to regulate the DNA repair genes among our signature did not show a consistent concomitant decrease in expression across both cell lines and time points (Figure 5A–5B). For example, after 24 hours of treatment, c-Myc and E2F1 increased in SUM149, but only E2F1 remained elevated after 72 hours of treatment. STAT3 was more consistently downregulated in SUM149, and this correlates with the fact that the known STAT3 target gene MDC1 was also robustly downregulated under the same conditions. In HCC1937 cells, c-Myc expression exhibited a biphasic response at both 24 and 72 hours, whereas E2F1 showed a biphasic response at the early time point but not at 72 hours. Once again STAT3 was most consistently downregulated in HCC1937 cells. The results indicate that expression of these factors may not reflect their activity; however, their target genes may be biomarkers of dinaciclib activity.

Because all of the DNA repair genes we examined were potently downregulated by 30 nM dinaciclib, we asked whether other G2/M arresting drugs also inhibit these pathways, or whether this signature is specific for dinaciclib. To examine this, we looked at three of the most differentially expressed genes from the dinaciclib-regulated candidates (BRCA1, MDC1, and RAD51) in SUM149 cells that were treated with MLN8237. MLN8237 (alisertib), is an Aurora kinase A inhibitor that is known to induce G2 arrest [33]. As expected, even a high dose of MLN8237 (1 μM), which was sufficient to induce a similar degree of cell proliferation inhibition, did not downregulate any of the DNA repair genes, and in the case of RAD51 increased its expression (Figure 5C and Supplementary Table 13). Hence, the dinaciclib-regulated gene signature is drug specific and not merely an indicator of altered cell cycle.

One consequence of unrepaired DNA damage in cells that have already committed to undergo cell division is polyploidy, which may lead to mitotic catastrophe [34]. We examined the polyploid population of cells in both IBC cell lines as well as in MDA-MB-468 and MDA-MB-436 cells as examples of antagonistic and synergistic cell lines. Cells were treated with sequential combinations of dinaciclib and epirubicin, and then cells were either collected or allowed to recover for 48 hours before fixation. We observed a negative correlation (r^2^ = 0.74) in the magnitude of polyploidy after treatment with the average CI values for each cell line (Figure 5D). These results suggested that cells that underwent the most polyploidy were most likely to have a synergistic response to combination treatment.

## DISCUSSION

This work for the first time identifies cyclin E as a biomarker for therapy in IBC. IBC currently lacks any targeted treatment strategies (apart from trastuzumab and pertuzumab for HER2-overexpressing tumors) and, due to its intrinsic aggressiveness, confers a poorer prognosis than other stage- and subtype-matched breast cancers. We found a universally high expression of cyclin E in a large cohort of IBC patient samples and demonstrated that the distribution of cyclin E immunophenotypes is distinct from that for our non-IBC cohort. In addition to the different distribution of subtypes in IBC, when we evaluated freedom-from-recurrence as a function of cyclin E phenotype, we found that cyclin E phenotype did not predict outcome in IBC, in contrast to the highly significant correlation between cytoplasmic cyclin E and poorer outcome in the non-IBC cohort.

Previous work from our group has shown that cytoplasmic LMW-E has novel kinase-dependent functions outside of the cell cycle that contribute to its oncogenicity, for example, as a regulator of EMT and cancer stem-cell phenotypes [11]. IBC is characterized by an early invasive phenotype, whereby tumor cells invade lymphovascular spaces as emboli, which results in the prototypical manifestation of IBC as a red engorged breast, and high incidence of *de novo* metastatic disease. Our work suggests that cyclin E may be a driver of this unique biology in IBC, and therefore early targeting of this pathway may be beneficial, especially in patients with demonstrated intrinsic resistance to chemotherapy. There is an ever-increasing body of work linking the quiescent nature of cancer stem cells with drug resistance, including a recent report in IBC, which showed that the cancer stem cell population (CD44hi, CD24low cells) expresses high levels of cyclin E, and combined targeting of CDK2 enhances response to chemotherapy [35]. However, this report used a very high dose of paclitaxel and concomitant treatment with a CDK2 inhibitor, versus our work which focuses on DNA damaging agents.

Cyclin E is an attractive target in IBC for a number of reasons. Our data from patient samples indicated a high incidence of overexpression, making biomarker selection as a criterion for the clinical trial unnecessary. Given IBC patient volumes in any single center, the feasibility of a biomarker-selected trial is a challenge if only a portion of the overall potential population would be eligible for the trial based on a lower incidence of the biomarker. Second, cyclin E is clearly an oncogenic driver in breast cancer, and it influences a number of important phenotypes beyond cell cycle progression such as EMT and cancer stem cell enrichment, which are prominent factors in IBC as well. Third, there are clinically available agents that target the cyclin E/CDK2 complex. One of these agents, dinaciclib, has caused unexpected high toxicity in early clinical trials, mainly due to the high doses used. In the first-in-human phase 1 trial, weekly dosing of dinaciclib was explored and the recommended dose for further investigation was considered to be 12 mg/m^2^ (resulting in a peak dose of approximately 1.2 μmol at 2 hours post-infusion) [36]. However, two subsequent clinical trials in which combinations of dinaciclib and other chemotherapies were administered (both in the metastatic breast cancer setting) used either 20 mg/m^2^ [37] or 50 mg/m^2^ [38]. Both resulted in closure of the studies due to extreme toxicity or minimal increases in progression-free survival. Future studies of dinaciclib should be combination studies but at substantially lower doses that are less likely to cause dose-limiting neutropenia, therefore allowing metronomic dosing on a weekly or more frequent schedule.

For the three ongoing clinical trials with publically available information about dosing, the doses being used are 7 and 9 mg/m^2^, which should be more tolerable than the doses in previous trials. For IBC specifically, our radiosensitization data argue that dinaciclib (or future CDK2 inhibitors that also downregulate DNA repair pathways) may be worthwhile drugs to combine with post-mastectomy radiation, particularly for women with TN-IBC with substantial residual disease, whom we know from retrospective data have a high recurrence risk even with comprehensive radiation.

We identified a number of DNA repair genes that are significantly inhibited following dinaciclib treatment. These corresponding proteins participate in several DNA repair pathways, including homologous recombination and mismatch repair. Several of these proteins are found within the same protein complexes (e.g., FANCA and BRCA1, and BRCA1 and BARD1). Depletion of multiple components of DNA damage recognition complexes and scaffolding proteins is likely sufficient to cause functional deficiency in the repair of double-strand breaks and interstrand crosslinks. In addition, this multifaceted inhibition of repair pathways likely leads to the inability to compensate by using alternative repair mechanisms, leaving cells with unrepaired DNA damage leading to cell death. Because dinaciclib inhibits transcriptional CDKs (CDK5 and CDK9), it is possible that the synergy results from inhibiting these proteins rather than CDK2 directly, a question worth exploring if more specific CDK2, CDK5, or CDK9 inhibitors are developed in the future.

We also found that STAT3 transcript levels decreased consistently as well as MDC1, which is a known target gene that is an important regulator of the ATM-Chk2 pathway [39]. STAT3 is hyperactivated in IBC, as demonstrated by a high incidence of phosphorylated STAT3 in tumor tissues and also elevated upstream cytokines such as IL6 known to activate this pathway [40]. There is a known link between induction of DNA damage and activation of CDK5, resulting in STAT3 phosphorylation and accelerated DNA repair [41]. CDK5 has also been linked to the DNA damage response via phosphorylation of ATM, which is required for ATM autophosphorylation and therefore its activity as a damage sensor [42]. Together these connections strongly suggest the potential for dinaciclib to not only transcriptionally regulate DNA damage response proteins but also do so via post-transcriptional regulation.

In summary, our data implicate cyclin E as a master regulator of a number of pathways important in the biology of IBC and resistance to chemotherapy. With these pathways in mind, future clinical trials of CDK inhibitors may be fruitful areas of development for IBC patients.

## MATERIALS AND METHODS

### Cell lines

A comprehensive list of the cell lines and their growth media is found in Supplementary Table 1. The sources of the cells and culture methods are given in the supplemental methods. Cells were mycoplasma tested and were authenticated upon receipt by short-tandem repeat (STR) profiling by the Characterized Cell Line Core Facility at The University of Texas MD Anderson Cancer Center, and cells from frozen vials were maintained in culture no more than 6 weeks.

### Patient samples and chart review

The sample collection, chart review, and data analysis for this research were approved by the Institutional Review Board of MD Anderson Cancer Center. All patient samples were from patients who provided informed consent for the banking/use of their tissue for research.

For outcome analysis, we utilized freedom-from-recurrence (FFR) as our endpoint, which is a modification of the recurrence-free survival endpoint from the guidelines of Hudis et al. [43] and was computed as time from diagnosis to date of first recurrence (local/regional or distant) or to last follow-up (if no recurrence). FFR captures only recurrences and does not include deaths as events, regardless of cause of death. Patients not experiencing the endpoint were censored at last follow-up.

### Cyclin E immunohistochemistry

Cyclin E immunohistochemical analysis was performed using optimized conditions as reported in Karakas et al. [16]. The primary antibody used was cyclin E (C-19, Santa Cruz Biotechnology, sc-198), which was diluted 1:1000. The scoring system was described in the original publication.

### Western blot analysis

Western blot analyses were performed as previously described [44] with the following modifications. The cell pellet was lysed in RIPA buffer with a cocktail of protease/phosphatase inhibitors (250 μg/ml leupeptin, 250 μg/ml aprotinin, 100 μg/ml pepstatin, 1 mM benzamidine, 100 μg/ml soybean trypsin inhibitor, 5.0 mM phenylmethylsulfonyl fluoride, 50 mM sodium fluoride, and 0.5 mM sodium orthovanadate). The primary antibodies used were cyclin E (HE12 monoclonal, Santa Cruz Biotechnology, sc-247), phospho-CDK2 (Thr160) (polyclonal, Cell Signaling, #2561), CDK2 (M2 polyclonal, Santa Cruz Biotechnology, sc-163), PARP (polyclonal, Cell signaling #9542), Caspase 3 (polyclonal, Cell Signaling #9662), Mcl1 (polyclonal, Santa Cruz Biotechnology, sc-819) and β-actin (monoclonal, Millipore, MAB1501R).

### High throughput survival assay (HTSA)

The timeline for this assay is shown as a schematic in Figure 3A. The details of assay development are found in the Supplementary methods and Supplementary Figure 1.

### Apoptosis assay

Apoptosis was quantified using annexin V/propidium iodide (PI) staining (Life Technologies, Foster City, CA, catalogue #V13241) and analyzed by flow cytometry according to the manufacturer's instructions. Washed cells were incubated with Alexa-488–conjugated annexin V antibody and 1 μg/mL PI for 15 minutes in the dark at room temperature. The stained cells were analyzed using a FACSCalibur flow cytometer and the CellQuest Pro Software, version 6.0.2 (BD Biosciences, Franklin Lakes, NJ).

### Cell cycle analysis

Nuclear DNA content was analyzed to determine the cell cycle phase of treated cells. Cells were harvested by trypsinization and fixed in 70% ethanol (in PBS). Following fixation and washing with PBS, cells were stained with 1 μg/mL PI in buffer overnight. The staining buffer consisted of PBS + 0.5% Tween-20 and 0.5% bovine serum albumin with 20 μg/mL RNase A. A FACSCalibur flow cytometer was used with data generated using CellQuest Pro Software, version 6.0.2 (BD Biosciences).

### RNA extraction and qRT-PCR

RNA was isolated from cells using the RNeasy mini kit (Qiagen, Valencia, CA). After isolation, the RNA was ethanol precipitated and resuspended at a concentration of 1 μg/μL. cDNA was made using the High Capacity First Strand cDNA Synthesis Kit (Life Technologies, catalogue #4368814) using 2 μg of total RNA per reaction. Quantitative PCR reactions were run using SYBR Green PCR master mix (Life Technologies, catalogue #4309155) on an AB7500 Fast Real-Time PCR machine. Relative expression of each gene was calculated using the ΔΔCT method, and GAPDH was used for normalization. Each reaction was performed in triplicate, and the experiments were repeated in duplicate or triplicate as indicated in the figure legends. Sequences of the primers used are found in the Supplementary methods.

## SUPPLEMENTARY MATERIALS AND METHODS, FIGURES AND TABLES



## Abbreviations

ATM: Ataxia-telangiectasia mutated
BRCA1: BRCA1, DNA repair associated gene
BRCA2: BRCA2, DNA repair associated gene
CDK2: Cyclin-dependent kinase 2
CI: Combination index
DNA: Deoxyribonucleic acid
FANCA: Fanconi anemia complementation group A
FFR: Freedom-from-recurrence
HTSA: High-throughput survival assay
IBC: Inflammatory breast cancer
LMW-E: Low-molecular weight isoform of cyclin E
MDC1: Mediator of DNA damage checkpoint 1
MSH2: mutS homolog 2
RAD51: RAD51 recombinase
STAT3: Signal transducer and activator of transcription 3
TNBC: Triple-negative breast cancer
TN-IBC: Triple negative inflammatory breast cancer
