# Supplementary material for: Cyclin E overexpression as a biomarker for combination treatment strategies in inflammatory breast cancer
Source: Oncotarget. 2017 Jan 17;8(9):14897–911. doi: 10.18632/oncotarget.14689 (PMC5362453; doi:10.18632/oncotarget.14689)
Supplement: Supplementary file 1 [file oncotarget-08-14897-s001.pdf]

Cyclin E overexpression as a biomarker for combination treatment strategies in inflammatory breast cancer

Supplementary Materials

SUPPLEMENTARY METHODS

Reagents

Dinaciclib was obtained from Merck, and a concentrated stock solution was prepared in 20% 2-hydroxylpropyl-beta-cyclodextrin (in water). Further dilutions were made in DMSO. Epirubicin was obtained from the pharmacy at The University of Texas MD Anderson Cancer Center already in solution in the hydrochloride form at a concentration of 2 mg/ml. Further dilutions were made in sterile water. Carboplatin was purchased from Sigma and dissolved in PBS at 10 mg/ml. Paclitaxel was purchased from Sigma and dissolved in DMSO. Meriolin 5 was a kind gift from Laurent Meijer (ManRos Therapeutics) and was dissolved in DMSO. MLN8237 was purchased from Selleck Chemicals and dissolved in DMSO. All drugs were stored at –20°C for long-term storage except for epirubicin, which was kept in the dark at 4°C as per package directions. The MTT was purchased from RPI Corporation and dissolved in serum-free MEM medium at a stock concentration of 2.5 mg/ml. This solution was filtered and stored in the dark at 4°C.

Description of patient cohorts

We used four cohorts of breast cancer patients and two cohorts of IBC patients for our study comparing the expression of cyclin E. Some of these samples have been previously reported upon in our manuscript describing the validation of cyclin E antibodies and relationship with outcome [1]. In total, the non-IBC cohort of 2510 samples was derived from four sources: 1) The first set included 725 patients with early-stage breast cancer (stage I–II) who underwent surgery at MD Anderson Cancer Center between 1985 and 1999. These tissues were analyzed using tissue microarrays with 1-mm biopsy cores. 2) The second set was purchased from the National Cancer Institute (NCI) Cancer Diagnosis Program and was a collection of samples from 951 patients with stage I–II breast cancer who underwent surgery between 1985 and 1997 at four different hospitals around the United States (Kaiser Permanente in Portland, OR; Washington University in St Louis, MO; Fox Chase Cancer Center in Philadelphia, PA; and the University of Miami in Miami, FL). Tissue microarrays from these samples were generated at the University of Virginia using 0.6-mm cores. 3) The third set consisted of tissue microarrays from 515 older patients with stage I-II disease treated at Nottingham University Hospitals, Nottingham, England, between 1987 and 2005. 4) The fourth set of samples (totaling 319 samples) was collected prospectively from MD Anderson under an IRB-approved protocol to specifically examine the relationship between cyclin E and other clinical factors related to prognosis in patients treated between 2000 and the present. For all cohorts, formalin-fixed and paraffin-embedded tissues were collected, and clinical characteristics were collected by manual review of charts or from MD Anderson research databases in the Departments of Surgical Oncology and Breast Medical Oncology. All of the 147 IBC patients with cyclin E results and at least one item of follow-up post-initial consult were from MD Anderson, but not all patients received all therapy at MD Anderson. Charts and correspondence from outside physicians were reviewed to complete the clinical variables table.

Cell culture conditions

SUM149 and SUM185 cell lines were obtained from Asterand, KPL4 cells were obtained from Naoto Ueno (MD Anderson), and the remaining cell lines were purchased from American Type Culture Collection (ATCC). Cells were maintained in a humidified incubator at 37°C in a 6.5% CO_2 atmosphere, with the medium changed every 2–3 days to maintain cell health.

High-throughput survival assay development

The high-throughput survival assay was developed to increase the capacity for screening drug combinations in many cell lines versus classic clonogenic assays. HTSA and clonogenic assays in side-by-side experiments are highly concordant [2] .

Cell density was optimized by plating a range of densities (usually 500–5000, but some were at a lower density) and following cell growth by performing MTT assays every other day. MTT assays were performed by diluting the stock concentration by 5-fold in the medium in which the cells usually grew. Medium from the plates was aspirated, and then the diluted MTT was added for a 4-hour incubation back in the 37°C incubator. The diluted MTT was then aspirated and the converted MTT crystals were solubilized in an 86% isopropanol solution containing 1% sodium dodecyl sulfate (SDS) and 0.04 M

hydrochloric acid. Characteristics of ideal densities are that they allow logarithmic growth throughout the experiment, and the absorbance of the wells remained within the linear range of the plate reader (Epoch reader, BioTek).

Prior to combination assays, the single drug dose-response curves were used to derive $IC_{10}/IC_{25}/IC_{50}/IC_{60}/IC_{75}$ values for 24-hour or 48-hour treatments, performed in duplicate and averaged. Each plate in the combination treatment experiments contained two columns of cells that were left untreated, allowing internal controls to be set to 100% to derive the survival fraction. In addition, two columns contained cells just treated with drug A, and the other eight columns were different concentrations of drug A and drug B. The drug A concentrations were the IC_{25}, IC_{50}, or IC_{75} values and the drug B concentrations ranged from IC_{10} through IC_{60}. After drug treatments, the old medium was replaced with fresh medium every 48 hours until the end of the experiment. CalcuSyn software was used to generate combination indexes from these data. The schematic for HTSA is provided in Supplementary Figure S1.

Quantitative real-time PCR primers

The sequences of the primers used were as follows:

ACTIN Fwd - CATGTACGTTGCTATCCAGGC
ACTIN Rev - CTCCTTAATGTCACGCACGAT
ATM Fwd - ATCTGCTGCCGTCAACTAGAA
ATM Rev - GATCTCGAATCAGGCGCTTAAA
BARD1 Fwd - CTGCTCGCGTTGTACTAACAT
BARD1 Rev - TCCAATGCAGTCACTTACACAAT
BRCA1 Fwd - ACCTTGGAACTGTGAGAACTCT
BRCA1 Rev - TCTTGATCTCCCACACTGCAATA
BRCA2 Fwd - ACAGAACCAATAAGCCATGTGG
BRCA2 Rev - TGCAAACATTAACGCAGCTTTC
C-MYC Fwd - GTCAAGAGGCGAACACACAAC
C-MYC Rev – TTGGACGGACAGGATGTATGC
E2F1 Fwd - CATCCCAGGAGGTCACTTCTG
E2F1 Rev - GACAACAGCGGTTCTTGCTC
FANCA Fwd - TTTGCTTGAGGTAGAAGGTCCA
FANCA Rev - CCCGGCTGAGAGAATACCCA
MDC1 Fwd - GGGCGGCTACATATCTTTAGTG
MDC1 Rev - GGCATTCGGCCTACCACATT
MSH2 Fwd - CACTGTCTGCGGTAATCAAGT
MSH2 Rev - CTCTGACTGCTGCAATATCCAAT
RAD51 Fwd - CAACCCATTTCACGGTTAGAGC
RAD51 Rev - TTCTTTGGCGCATAGGCAACA
STAT3 Fwd - ACCAGCAGTATAGCCGCTTC
STAT3 Rev - GCCACAATCCGGGCAATCT

Statistics and software

All experiments were performed in at least triplicate unless otherwise indicated in the figure legends or text. Bar graphs show the mean of all experiments pooled together, and error bars signify standard deviation from the mean. For comparisons, unpaired two-sided *t*-tests or ANOVAs (for more than two samples) were used, and $P < 0.05$ was the cut-off for significance.

Numerical data were processed using Excel for simple calculations and normalization. Final versions of graphs were made using GraphPad Prism 6 software, and *t*-tests and ANOVA were also performed using Prism. CalcuSyn software (Biosoft, Cambridge, UK) was used to generate combination indexes as described under HTSA. Heatmaps were generated using Tableau Desktop professional, version 9.3.4 (Tableau Software, Seattle, WA).

REFERENCES

1. Karakas C, Biernacka A, Bui T, Sahin AA, Yi M, Akli S, Schafer J, Alexander A, Adjapong O, Hunt KK, Keyomarsi K. Cytoplasmic Cyclin E and Phospho-Cyclin-Dependent Kinase 2 Are Biomarkers of Aggressive Breast Cancer. Am J Pathol. 2016; 186:1900–12.
2. Nanos-Webb A, Jabbour NA, Multani AS, Wingate H, Oumata N, Galons H, Joseph B, Meijer L, Hunt KK, Keyomarsi K. Targeting low molecular weight cyclin E (LMW-E) in breast cancer. Breast Cancer Res Treat. 2012; 132:575–88.

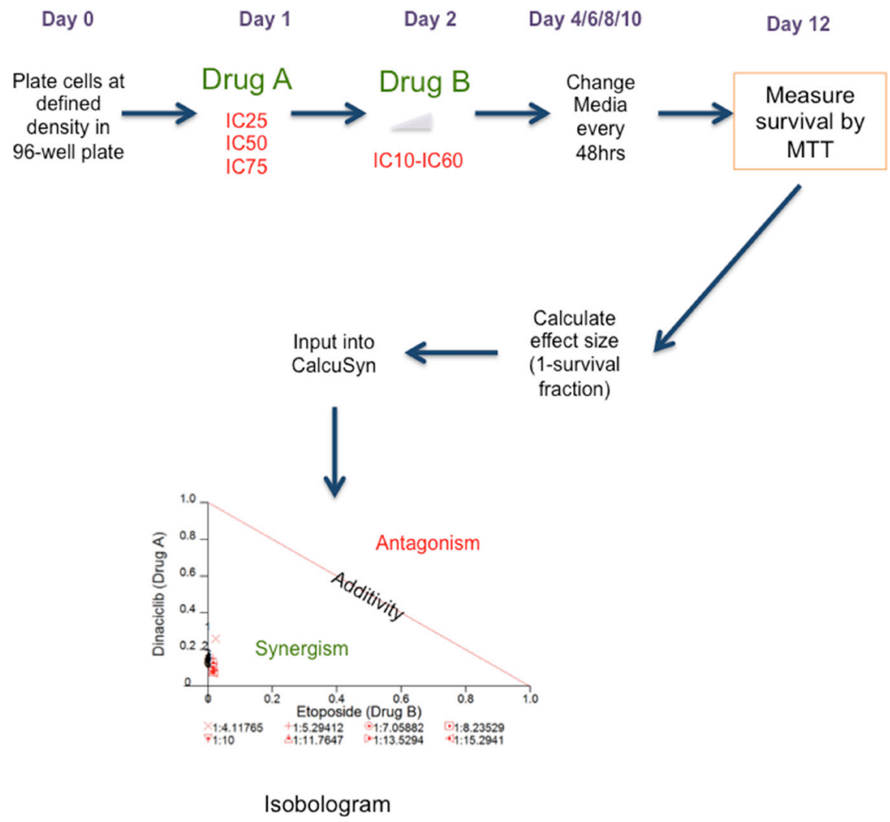

Supplementary Figure 1: Detailed protocol schematic for the high throughput survival assay (HTSA). Cells were treated with drug A at three different concentrations for 24 hours; drug A was removed and cells were treated with drug B (IC_{10}-IC_{60}) for 48 hours followed by a change of fresh drug-free medium every other day until day 12, when survival was measured. CalcuSyn software was then used to calculate the combination index (CI) and generate isobolograms (graphs of isoeffect plots). Each axis represents the dose of one drug, and each dot is the magnitude of the effect of a dose pair.

A

p53 Mutation status	Cell Line	Dina-Epi	Epi-Dina	Dina-Carbo	Carbo-Dina
		Sequence			
WT	MCF7	1.019	0.602	0.819	1.159
	KPL4	0.956	0.911	0.927	0.945
HET	T47D	0.976	1.160		
MT	BT549	0.781	1.216	0.879	1.100
	HCC1806	0.899	0.621	0.762	1.214
	HCC1937	0.441	1.111	0.733	1.025
	MDA-MB-157	1.254	1.023	0.482	0.980
	MDA-MB-231	0.922	1.054		
	MDA-MB-361	0.661	1.100	0.462	1.099
	MDA-MB-436	0.837	1.114	0.634	0.748
	MDA-MB-468	1.108	1.309	0.646	0.794
	SKBR3	1.089	0.895	1.014	0.802
	SUM149	0.771	1.057	0.761	0.840
	SUM185	0.953	1.078	1.561	0.695

Combination Index: 0.441 – 1.561

Rb Mutation Status	Cell Line	Dina-Epi	Epi-Dina	Dina-Carbo	Carbo-Dina
		Sequence			
WT	HCC1806	0.899	0.621	0.762	1.214
	HCC1937	0.441	1.111	0.733	1.025
	KPL4	0.956	0.911	0.927	0.945
	MCF7	1.019	0.602	0.819	1.159
	MDA-MB-157	1.254	1.023	0.482	0.980
	MDA-MB-231	0.922	1.054		
	MDA-MB-361	0.661	1.100	0.462	1.099
	SKBR3	1.089	0.895	1.014	0.802
	SUM149	0.771	1.057	0.761	0.840
	SUM185	0.953	1.078	1.561	0.695
	T47D	0.976	1.160		
MT	BT549	0.781	1.216	0.879	1.100
	MDA-MB-436	0.837	1.114	0.634	0.748
	MDA-MB-468	1.108	1.309	0.646	0.794

Combination Index: 0.441 – 1.561

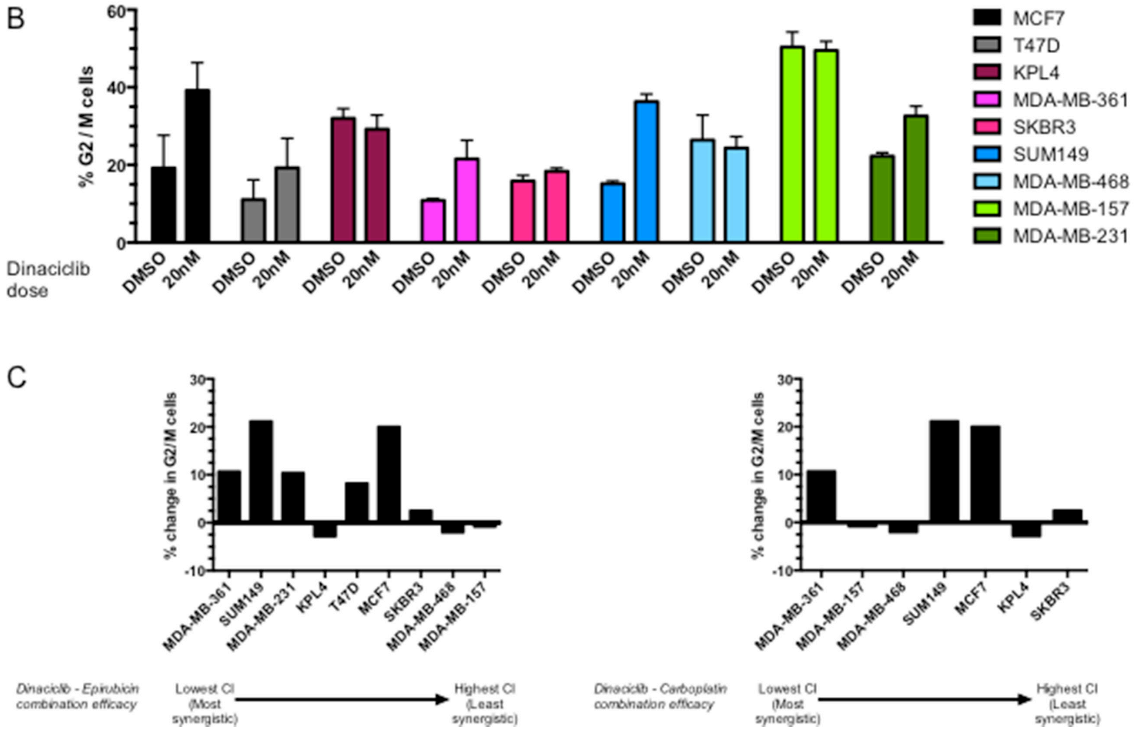

Supplementary Figure 2: Analysis of factors potentially associated with dinaciclib-induced synergy. (**A**) Heatmaps of combination drug screening (Figure 4A) presented as a function of p53 mutation status (left) or Rb mutation status (right). Similar to the heatmap in the main figure, the numbers indicate average CIs for the best plate (most synergistic) for each sequence. (**B**) Proportion of G2/M cells in the same samples as for Figure 4B. Cells were treated with 20 nM dinaciclib or DMSO control for 48 hours and fixed for propidium iodide staining. Cells are color coded by molecular subtype using different shades of the main color, i.e., basal TNBC cells are blue, mesenchymal TNBC cells are green, HER2+ cells are red/orange, and luminal (HER2-negative) cells are shades of grey. (**C**) Change in G2/M proportion after treatment in cells, ordered from lowest CI to highest CI for dinaciclib followed by epirubicin (left) or carboplatin (right). No correlation seemed to exist between the magnitude of cell cycle arrest by dinaciclib and subsequent synergy.

Supplementary Table 1: Cell line culture media

Cell line	Medium	
SUM149	Ham’s F12 + 5% FBS + 5 µg/ml insulin + 1 µg/ml hydrocortisone	*IBC
HCC1806	Alpha MEM + 10% FBS + additions	
HCC1937	Alpha MEM + 10% FBS + additions	
MDA-MB-468	DMEM + 10% FBS	
BT-549	RPMI 1640 + 10% FBS + 1 µg/ml insulin	
MDA-MB-157	Alpha MEM + 10% FBS + additions	
MDA-MB-231	Alpha MEM + 10% FBS + additions	
MDA-MB-436	Alpha MEM + 10% FBS + additions	
SUM185	Hams F12 + 5% FBS + 5 µg/ml insulin + 1 µg/ml hydrocortisone	
KPL4	DMEM/F12 (50:50) + 10% FBS	*IBC
SKBR3	Alpha MEM + 10% FBS + additions	
MDA-MB-361	DMEM + 10% FBS + 1 mM sodium pyruvate	
MCF7	Alpha MEM + 10% FBS + additions	
T47D	Alpha MEM + 10% FBS + additions	

Alpha MEM additions:

1 µg/ml Insulin
1 µg/ml Hydrocortisone
25 ng/ml Epidermal growth factor (EGF)
1 mM Sodium pyruvate
1× Non-essential amino acids
2 mM L Glutamine
10 mM HEPES

Supplementary Table 2: Plating densities used for HTSA experiments

Cell line	Density	
SUM149	3500	*IBC
HCC1806	500	
HCC1937	500	
MDA-MB-468	1500	
BT-549	2000	
MDA-MB-157	500	
MDA-MB-231	500	
MDA-MB-436	4000	
SUM185	4500	
KPL4	1500	*IBC
SKBR3	1500	
MDA-MB-361	4500	
MCF7	100	
T47D	1000	

The cell numbers refer to cells plated in a 96-well plate.

Supplementary Table 3: Doses of dinaciclib used for HTSA experiments

Cell line	24 hours (drug A)			48 hours (drug B)		
	IC_{25}	IC_{50}	IC_{75}	IC_{10}	IC_{60}	
SUM149	4	8	17	2	10	*IBC
HCC1806	6.04	15.5	36.73	3.59	26.04	
HCC1937	3.75	12.28	33.05	3.98	20.37	
MDA-MB-468	4	9	18	3	13	
BT-549	7.29	15.98	35.65	4.03	20.25	
MDA-MB-157	2.34	7.59	30.9	1.48	7.41	
MDA-MB-231	1.26	4.57	22.91	0.5	4.79	
MDA-MB-436	2.02	4.67	12.52	0.96	5.82	
SUM185	3.09	4.24	17.96	1.03	10.59	
KPL4	6.02	7.24	10.71	5	23	*IBC
SKBR3	3.77	7.48	18.09	1.58	6.72	
MDA-MB-361	10.21	27.57	105.24	4.24	24.22	
MCF7	3.12	6.76	18.64	1.86	7.75	
T47D	20.72	55.27	200.67	3.48	25.71	

All concentrations are nanomolar values (nM).

Supplementary Table 4: Doses of epirubicin used for HTSA experiments

Cell line	24 hours (drug A)			48 hours (drug B)		
	IC_{25}	IC_{50}	IC_{75}	IC_{10}	IC_{60}	
SUM149	11	21	32	6	23	*IBC
HCC1806	12.19	23.01	34.33	7.68	24.09	
HCC1937	7.3	14.07	22.92	1.84	14.96	
MDA-MB-468	4.94	7.17	12.23	3.98	7.51	
BT-549	7.68	14.49	24.65	4.08	16.17	
MDA-MB-157	6.6	13.48	23.6	1.47	17.14	
MDA-MB-231	9.89	16.8	35.91	5.19	14.1	
MDA-MB-436	1.92	4.4	9.83	0.76	3.59	
SUM185	20.75	35.53	37.45	5.06	19.52	
KPL4	15	23	32	4	16	*IBC
SKBR3	9.8	28.4	45.01	3.28	27.62	
MDA-MB-361	18.19	50.45	168.78	9.8	42.76	
MCF7	11.44	21.7	32.04	1.03	17.99	
T47D	5.85	12.91	23.19	10.94	26.75	

All concentrations are nanomolar values (nM).

Supplementary Table 5: Doses of carboplatin used for HTSA experiments

Cell line	24 hours (drug A)			48 hours (drug B)		
	IC_{25}	IC_{50}	IC_{75}	IC_{10}	IC_{60}	
SUM149	1.11	2.256	6.274	0.379	1.964	*IBC
HCC1806	2.84	7.648	18.546	1.617	9.097	
HCC1937	2.587	5.673	12.924	0.978	5.288	
MDA-MB-468	0.52	1.369	3.566	0.239	1.608	
BT-549	3.421	6.707	11.34	1.239	4.579	
MDA-MB-157	7.113	14.511	29.864	0.962	8.611	
MDA-MB-436	0.067	0.1347	0.2694	0.0269	0.2694	
SUM185	3.367	5.387	10.774	0.808	5.387	
KPL4	10.33	23.67	45.548	3.84	22.91	*IBC
SKBR3	0.525	1.47	4.35	0.089	1.095	
MDA-MB-361	6.97	17.023	39.853	1.67	10.75	
MCF7	3.621	7.698	17.683	0.195	4.24	

All concentrations are micromolar values (μM).

Supplementary Table 6: Doses of etoposide used for HTSA experiments

Cell line	24 hours (drug A)			48 hours (drug B)		
	IC_{25}	IC_{50}	IC_{75}	IC_{10}	IC_{60}	
SUM149	0.16	0.36	0.69	0.07	0.26	*IBC
KPL4	0.37	0.98	2.43	0.16	0.93	*IBC

All concentrations are micromolar values (μM).

Supplementary Table 7: Doses of paclitaxel used for HTSA experiments

Cell line	24 hours (drug A)			48 hours (drug B)		
	IC_{25}	IC_{50}	IC_{75}	IC_{10}	IC_{60}	
SUM149	1	2	5	1	3	*IBC
KPL4	1	3	8	1	3	*IBC

All concentrations are nanomolar values (nM).

Supplementary Table 8: Doses of meriolin 5 used for HTSA experiments

Cell line	24 hours (drug A)			48 hours (drug B)		
	IC_{25}	IC_{50}	IC_{75}	IC_{10}	IC_{60}	
SUM149	14.99	34.33	62.12	7.59	28.51	*IBC
KPL4	29.02	62.57	100.01	3.32	25.87	*IBC

All concentrations are nanomolar values (nM).

Supplementary Table 9: Entire dataset for dinaciclib-epirubicin HTSAs

Cell line	Dinaciclib-carboplatin			Carboplatin-dinaciclib			
	IC_{25}	IC_{50}	IC_{75}	IC_{25}	IC_{50}	IC_{75}	
SUM149	1.499	1.637	0.7605	0.9282	0.9314	0.8404	*IBC
HCC1806	1.422	0.7623	1.211	1.255	1.214	1.326	
HCC1937	1.336	1.025	0.7334	1.041	1.025	1.285	
MDA-MB-468	1.411	1.183	0.646	1.122	1.097	0.7944	
BT-549	2.071	0.8793	1.02	1.148	1.25	1.1	
MDA-MB-157	1.472	1.399	0.4816	1.085	0.9801	1.035	
MDA-MB-436	1.038	1.26	0.6341	0.8144	0.8392	0.7479	
SUM185	1.561	1.605	1.069	0.6951	0.7959	0.8994	
KPL4	1.219	1.181	0.9274	1.26	0.9453	1.046	*IBC
SKBR3	1.14	1.061	1.014	0.9376	0.8374	0.8018	
MCF7	1.256	1.233	0.8186	1.18	1.167	1.159	

The values are average combination indexes for two or three independent experiments for each direction.

Supplementary Table 10: Entire dataset for dinaciclib-carboplatin HTSAs

Cell line	Dinaciclib-carboplatin			Carboplatin-dinaciclib			
	IC_{25}	IC_{50}	IC_{75}	IC_{25}	IC_{50}	IC_{75}	
SUM149	1.499	1.637	0.7605	0.9282	0.9314	0.8404	*IBC
HCC1806	1.422	0.7623	1.211	1.255	1.214	1.326	
HCC1937	1.336	1.025	0.7334	1.041	1.025	1.285	
MDA-MB-468	1.411	1.183	0.646	1.122	1.097	0.7944	
BT-549	2.071	0.8793	1.02	1.148	1.25	1.1	
MDA-MB-157	1.472	1.399	0.4816	1.085	0.9801	1.035	
MDA-MB-436	1.038	1.26	0.6341	0.8144	0.8392	0.7479	
SUM185	1.561	1.605	1.069	0.6951	0.7959	0.8994	
KPL4	1.219	1.181	0.9274	1.26	0.9453	1.046	*IBC
SKBR3	1.14	1.061	1.014	0.9376	0.8374	0.8018	
MCF7	1.256	1.233	0.8186	1.18	1.167	1.159	

The values are average combination indexes for two or three independent experiments for each direction.

Supplementary Table 11: Entire dataset for qRT-PCR on SUM149 cells treated with dinaciclib

Gene	24 hours				72 hours			
	DMSO	10 nM	20 nM	30 nM	DMSO	10 nM	20 nM	30 nM
ATM	1.00	1.00	0.95	0.43	1.00	0.76	0.32	0.23
BARD1	1.00	1.04	1.18	0.46	1.00	1.19	0.73	0.40
BRCA1	1.00	0.92	0.45	0.17	1.00	0.92	0.36	0.21
BRCA2	1.00	0.88	0.72	0.45	1.00	0.88	0.95	0.61
FANCA1	1.00	1.44	1.06	0.26	1.00	1.28	0.97	0.58
MDC1	1.00	0.77	0.44	0.17	1.00	0.74	0.58	0.42
MSH2	1.00	1.31	0.84	0.23	1.00	1.15	0.70	0.48
RAD51	1.00	1.49	0.96	0.35	1.00	1.41	0.63	0.49
C-MYC	1.00	1.18	1.40	3.70	1.00	0.51	0.17	0.48
E2F1	1.00	4.47	4.37	1.30	1.00	1.73	3.62	6.49
STAT3	1.00	0.87	0.62	0.46	1.00	1.27	0.70	0.44

These results correspond to the heatmap in the main Figure 5A. The values are fold changes relative to the DMSO control for the same time point.

Supplementary Table 12: Entire dataset for qRT-PCR on HCC1937 cells treated with dinaciclib

Gene	24 hours				72 hours			
	DMSO	10 nM	20 nM	30 nM	DMSO	10 nM	20 nM	30 nM
ATM	1.00	1.11	0.50	0.31	1.00	0.97	0.50	0.23
BARD1	1.00	1.05	0.67	0.19	1.00	1.01	0.80	0.20
BRCA1	1.00	0.92	0.15	0.08	1.00	0.69	0.36	0.11
BRCA2	1.00	0.92	0.44	0.47	1.00	0.90	0.76	0.56
FANCA1	1.00	1.31	0.34	0.18	1.00	1.24	0.54	0.17
MDC1	1.00	0.85	0.17	0.18	1.00	0.65	0.39	0.16
MSH2	1.00	0.94	0.34	0.32	1.00	1.12	0.57	0.29
RAD51	1.00	1.15	0.34	0.24	1.00	1.06	0.61	0.16
C-MYC	1.00	1.29	0.51	0.41	1.00	1.28	0.83	0.34
E2F1	1.00	1.25	0.23	0.26	1.00	0.88	0.37	0.15
STAT3	1.00	1.03	0.64	0.49	1.00	1.10	0.64	0.43

These results correspond to the heatmap in the main Figure 5B. The values are fold changes relative to the DMSO control for the same time point.

Supplementary Table 13: Entire dataset for qRT-PCR on SUM149 cells treated with 1 μM MLN8237

Gene	24 hours		72 hours	
	DMSO	MLN	DMSO	MLN
BRCA1	1.00	0.93	1.00	1.34
MDC1	1.00	0.99	1.00	1.27
RAD51	1.00	1.17	1.00	1.86

These results correspond to the heatmap in the main Figure 5C. The values are fold changes relative to the DMSO control for the same time point.
